# Hypoxia preconditioning of adipose stem cell-derived exosomes loaded in gelatin methacryloyl (GelMA) promote type H angiogenesis and osteoporotic fracture repair

**DOI:** 10.1186/s12951-024-02342-6

**Published:** 2024-03-15

**Authors:** Xiaoqun Li, Shuo Fang, Shaohai Wang, Yang Xie, Yan Xia, Panfeng Wang, Zichen Hao, Shuogui Xu, Yuntong Zhang

**Affiliations:** 1 Department of Orthopaedics, The First Affiliated Hospital of Navy Medical University, Shanghai, China; 2 Department of Plastic Surgery, The First Affiliated Hospital of Navy Medical University, Shanghai, China; 3 Department of Stomatology, Shanghai East Hospital, Tongji University School of Medicine, Shanghai, China

**Keywords:** Hypo-ADSC-Exos, miR-21-5p, Angiogenesis, GelMA, Type H vessels, Osteoporotic fracture repair

## Abstract

**Abstract:**

The challenges posed by delayed atrophic healing and nonunion stand as formidable obstacles in osteoporotic fracture treatment. The processes of type H angiogenesis and osteogenesis emerge as pivotal mechanisms during bone regeneration. Notably, the preconditioning of adipose-derived stem cell (ADSC) exosomes under hypoxic conditions has garnered attention for its potential to augment the secretion and functionality of these exosomes. In the present investigation, we embarked upon a comprehensive elucidation of the underlying mechanisms of hypo-ADSC-Exos within the milieu of osteoporotic bone regeneration. Our findings revealed that hypo-ADSC-Exos harboured a preeminent miRNA, namely, miR-21-5p, which emerged as the principal orchestrator of angiogenic effects. Through in vitro experiments, we demonstrated the capacity of hypo-ADSC-Exos to stimulate the proliferation, migration, and angiogenic potential of human umbilical vein endothelial cells (HUVECs) via the mediation of miR-21-5p. The inhibition of miR-21-5p effectively attenuated the proangiogenic effects mediated by hypo-ADSC-Exos. Mechanistically, our investigation revealed that exosomal miR-21-5p emanating from hypo-ADSCs exerts its regulatory influence by targeting sprouly1 (SPRY1) within HUVECs, thereby facilitating the activation of the PI3K/AKT signalling pathway. Notably, knockdown of SPRY1 in HUVECs was found to potentiate PI3K/AKT activation and, concomitantly, HUVEC proliferation, migration, and angiogenesis. The culminating stage of our study involved a compelling in vivo demonstration wherein GelMA loaded with hypo-ADSC-Exos was validated to substantially enhance local type H angiogenesis and concomitant bone regeneration. This enhancement was unequivocally attributed to the exosomal modulation of SPRY1. In summary, our investigation offers a pioneering perspective on the potential utility of hypo-ADSC-Exos as readily available for osteoporotic fracture treatment.

**Graphical Abstract:**

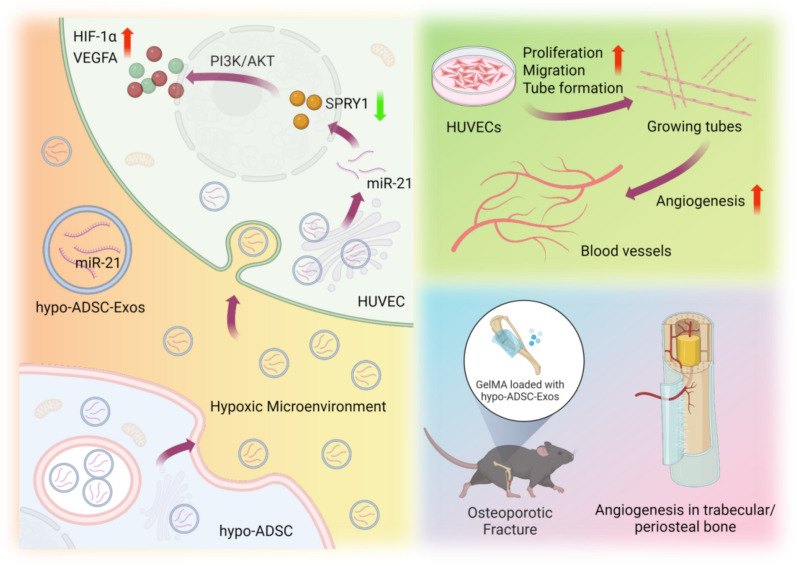

**Supplementary Information:**

The online version contains supplementary material available at 10.1186/s12951-024-02342-6.

## Introduction

Effective treatment for osteoporotic fracture remains challenging, and poor or impaired recovery from osteoporotic fracture is an increasingly severe problem in an ageing society [1–3]. In approximately 5–10% of cases, osteoporotic fracture union is delayed or compromised and can lead to re-operations and high socioeconomic costs [4–6]. Throughout the processes of osteoporotic bone regeneration and fracture healing, angiogenesis is indispensable [7]. Type H vessels is a special vascular subtype, charactered with positivity for CD31 and Endomucin (Emcn) in the vascular endothelia, it has recently been proved to couple angiogenesis with osteogenesis during the bone metabolism [7–10]. Meanwhile, the bone can modulate the formation of type H vessels through the secretion of various factors such as vascular endothelial growth factor (VEGF) and platelet-derived growth factor type BB (PDGF-BB) [8, 11]. Our previous study found that the number of H-type vessels was greatly reduced in callus tissue from osteoporotic fractures [11], and promoting the formation of type H vessels in the local fracture region has been proved to be a promising strategy to accelerate osteoporotic fracture healing [8, 12].

Exosomes are important nano-carriers that deliver genetic messages, such as proteins, enzymes, cytokines and miRNAs, ranging from 40 to 100 nm in diameter. These particles contain an abundance of bioactive molecules, which mediate intercellular communication in the body [13–15]. Exosomal treatment not only has the efficacy in stem cell transplantation, but also overcomes the risk of cell death, immunogenicity and tumorigenicity in the body [16]. Hypoxia refers to a decline of tissue oxygen level. Previous studies have demonstrated that, communications between the cells and the microenvironment may affect the contents of the exosomes and their physiological functions [17, 18]. The hypoxic microenvironment has been proved promote the expression of angiogenesis cytokines such as the hypoxia inducible factor (HIF) and vascular growth factor-A (VEGF-A), which enhanced the wound healing capacity [19, 20]. Exosomes from hypoxic pretreated ADSCs can accelerate the proliferation and migration of HUVECs and promote angiogenesis, and it was widely used in tissue regeneration, such as post-trauma osteoarthritis, diabetic wound healing and nerve damage repair [21–23]. However, the application of hypo-ADSC-Exos in bone regeneration has not been reported yet. Gelatin methacrylate (GelMA), derived from gelatin and capable of photopolymerization, has recently gained attention as a promising material for applications in tissue engineering [24–26]. Above all, GelMA loaded with hypoxic pre-treated ADSCs (hypo-ADSCs) are supposed to be an effective regimen to promote angiogenesis and for osteoporotic fracture treatment.

To better understand exosome-mediated intercellular communication between endothelial cells and hypo-ADSCs in angiogenesis, we investigated the critical miRNAs of hypo-ADSC-Exos and evaluated their roles in endothelial cell biological function. In addition, the target genes and molecular signaling pathways in recipient cells were revealed. Ultimately, we verified the hypothesized molecular mechanisms and demonstrated the possible optimal therapeutic effects of hypo-ADSC-Exos loaded in GelMA application in an osteoporotic fracture model.

## Results

### Identification of the exosomes derived from hypoxic ADSCs

**Fig. 1:**
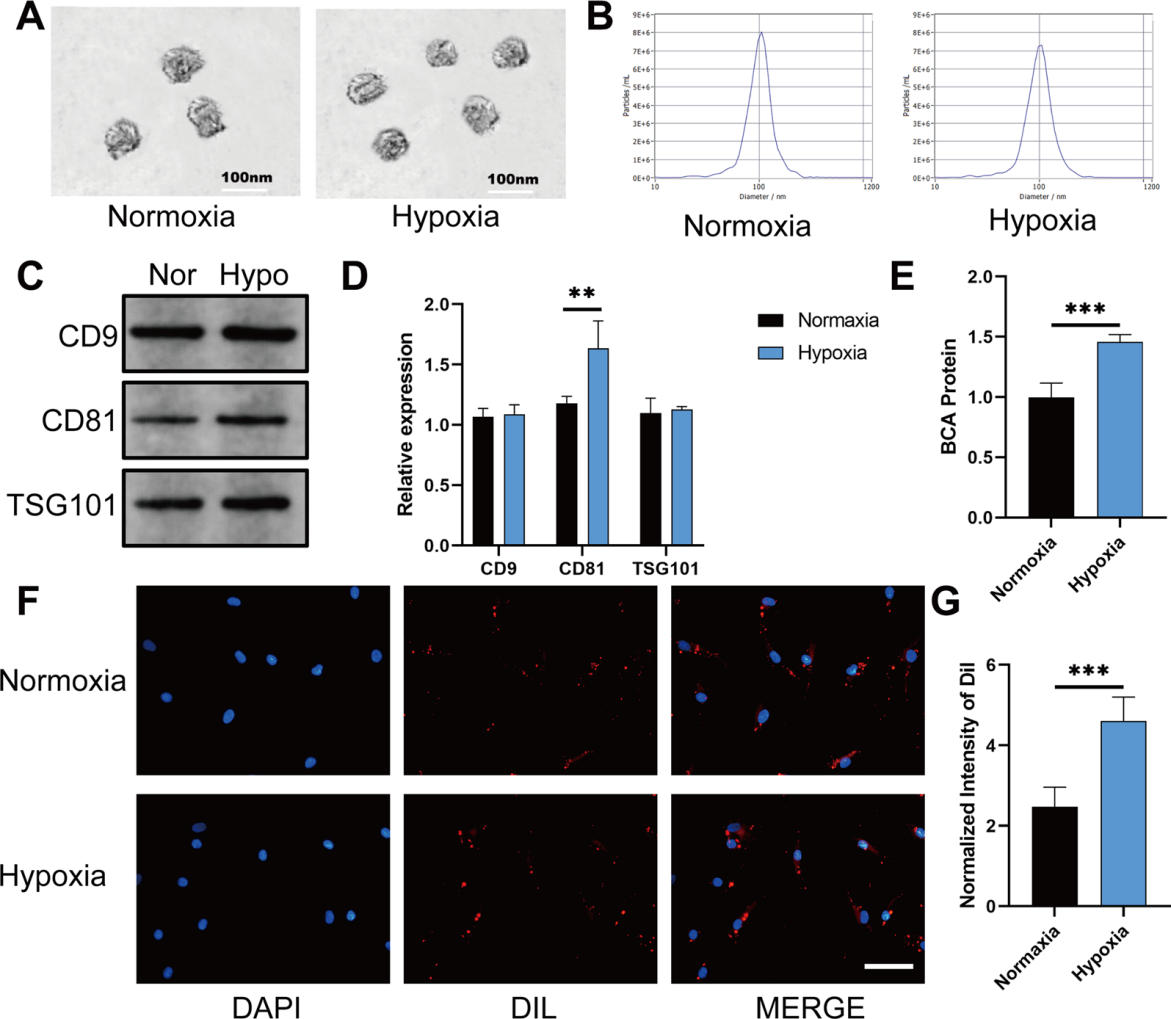
Identification of the exosomes derived from hypoxic ADSCs. **A** Morphology of each group of exosomes photographed via TEM. Scale bar: 100 nm. **B** Size distribution of the exosomes was determined with a NanoSight LM10 instrument. **C** Western blot of CD9, CD81 and TSG101 in the exosomes. **D** Analysis of the expression of CD9, CD63 and TSG101 in the exosomes. **E** Exosome protein concentration in the normoxia and hypoxia groups using the BCA assay. **F** Uptake of the red fluorescence dye Dil labelled Norm-Exos and Hypo-Exos into HUVECs. Scale bar = 20 μm. n = 5. **G** Statistical evaluation of fluorescence intensities in the two groups (*p < 0.05; **p < 0.01; ***p < 0.001)

The morphology of the purified exosomes was observed by using transmission electron microscopy (TEM). Whether exposed to the hypoxic conditions or not, all exosomes had a saucer-like shape with a diameter ranging from 40 to 100 nm (Fig. 1A). The diameter of the exosomes was determined with a NanoSight LM10 instrument (NanoSight, Amesbury, U.K., http://www.nanosight.com) (Fig. 1B). The markers of the exosomes, namely, CD9, CD81 and TSG101, were also detected by Western blotting, and the results showed that compared with the amounts in the UEFS, the CD9, CD81 and TSG101 levels were enriched in the exosome samples (Fig. 1C, D). In addition, the concentration of proteins in exosomes derived from different groups were detected, the results showed that, the hypo-ADSC-Exos had a higher concentration of proteins compared with the ADSC-Exos (Fig. 1E). Exosomes are nanoparticles secreted by the donor cells and taken up by the recipient cells. We further examined the uptake of hypo-ADSC-Exos and ADSC-Exos by HUVECs. Exosomes were dyed by Dil dye and co-cultured with HUVECs for 1 day. As shown in Fig. 1F, G the quantity of exosomes taken up by HUVECs was remarkably improved in the hypo-ADSC-Exos group (Fig. 1F, G). These findings indicated that the remodeling and extraction of the hypo-ADSC-Exos were effective and reliable, and hypoxic precondition made the exosomes easily taken up by HUVECs.

### Hypo-ADSC-Exos facilitate the proliferation, migration and tube formation of HUVECs in vitro

**Fig. 2:**
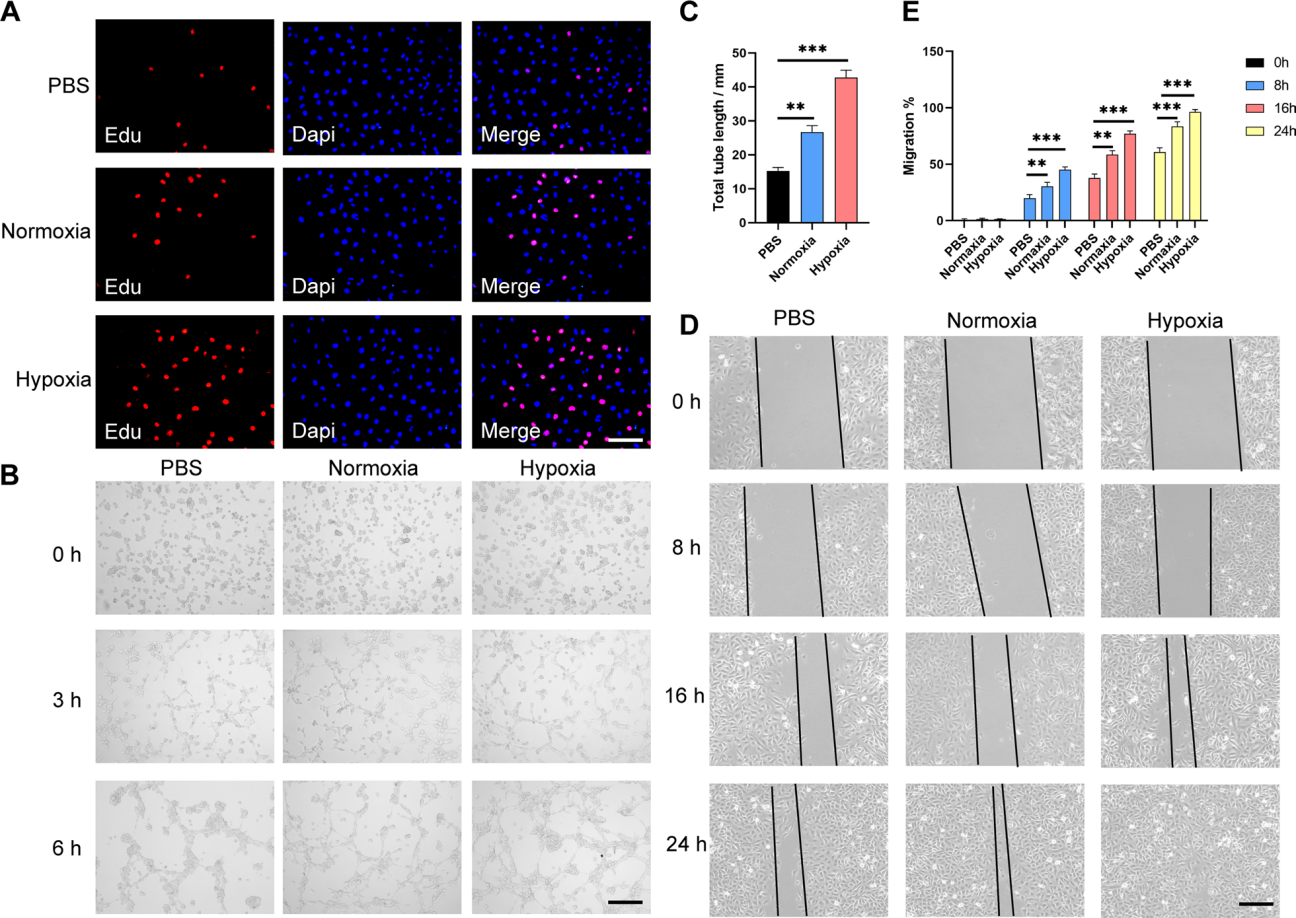
Hypo-ADSC-Exos promote the proliferation, migration, and tube formation in HUVECs in vitro. **A** Cell proliferation of HUVECs measured by EDU staining. Scale bar = 200 μm. n = 5. **B** Representative images of tube formation in HUVECs treated with PBS, Norm-Exos or Hypo-Exos. Scale bar = 200 μm. n = 5. **C** Quantitative analysis of the tube formation assay in the normoxia and hypoxia groups. **D** Extracted exosomes promoted cell migration as determined by a scratch test at 0 h, 8 h, 16 h and 24 h after treatment. n = 5. **E** Quantitative analysis of the migration rate of HUVECs (*p < 0.05; **p < 0.01; ***p < 0.001)

To explore the effects of hypo-ADSC-Exos on angiogenesis in vitro, we co-cultured the HUVECs with PBS solutions, hypo-ADSC-Exos and ADSC-Exos. The proliferation abilities of HUVECs were evaluated by the CCK-8 and EDU assays. The results indicated that, both ADSC-Exos and hypo-ADSC-Exos groups promoted the proliferation of HUVECs compared with the control group, it is worth noting that, the hypo-ADSC-Exos groups contributed to a significantly higher increase in the number of EDU-positive cells (Fig. 2A and Additional file 1: Fig. S1). The tube lengths were then analyzed by tube formation experiments at different time points. The results showed that, after co-cultured with exosomes for 6 h, both the ADSC-Exos and hypo-ADSC-Exos groups had accelerated the tube formation capability compared with the control group. However, the tube formation capability was more promoted by hypo-ADSC-Exos significantly (Fig. 2B, C). The effects of hypo-ADSC-Exos on cell migration were also examined. The results demonstrated that, both ADSC-Exos and hypo-ADSC-Exos groups promote the migration of HUVECs. However, the hypo-ADSC-Exos intervention remarkably enhanced the migration of HUVECs compared with the ADSC-Exos treatment (Fig. 2D, E). In conclusion, these results indicated that, the hypo-ADSC-Exos showed a greater capability on enhancing proliferation, tube formation and migration of HUVECs compared the ADSC-Exos in vitro.

### miR-21-5p was the most relevant miRNA in the Hypo-ADSC-Exos and was shuttled directly from Hypo-ADSCs to HUVECs via exosomes

**Fig. 3:**
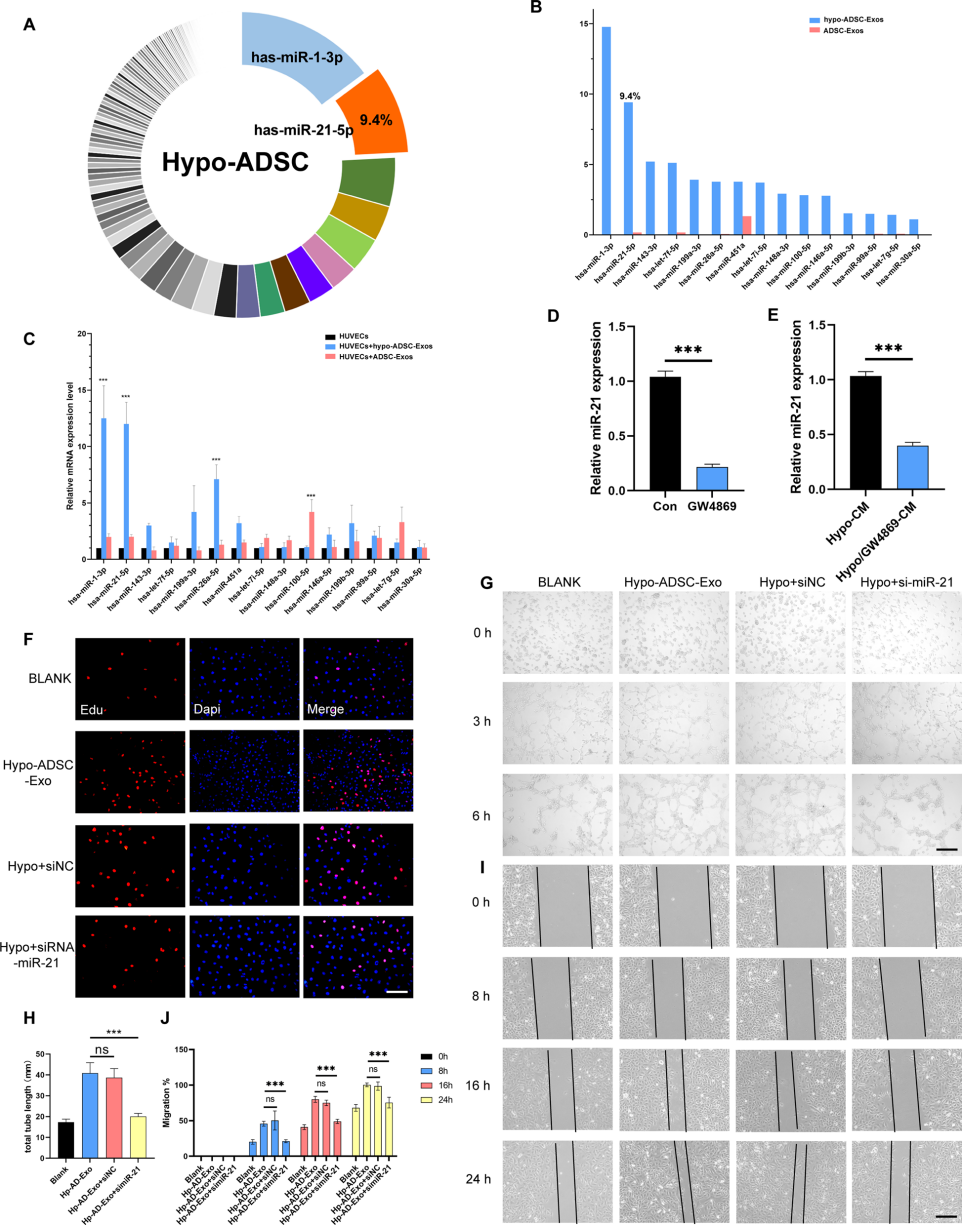
miR-21 is up-regulated in exosomes derived from the hypoxic ADSCs. **A** Pie chart of the exosomal miRNA abundance analysis by high-throughput small RNA sequencing. The top 15 most abundant miRNAs in Hypo-ADSC-derived exosomes are colour labelled. **B** Top 15 most abundant miRNA in the Hypo-ADSC-Exos and ADSC-Exos. **C** qRT-PCR results show the 15 most abundant miRNAs in different gourps of HUVECs subjected to interference for 48 h. **D** The expression of miR-21 in the CM of Hypo-ADSCs treated with GW4869 was significantly decreased compared with the control group. **E** Expression of miR-21 in the HUVECs treated with GW4869-CM for 48 h was also significantly decreased compared to that in the HUVECs treated with Hypo-ADSC-CM. **F** Cell proliferation of HUVECs measured by EDU staining. Scale bar = 200 μm. n = 5. **G** Representative images of tube formation in HUVECs treated with PBS, Hypo-Exos, Hypo-Exos+si-miR-NC and Hypo-Exos, Hypo-Exos+si-miR-21. Scale bar = 200 μm. n = 5. **H** Quantitative analysis of the tube formation assay in different groups. **I** Extracted exosomes promoted cell migration as determined by a scratch test at 0 h, 8 h, 16 h and 24 h after treatment. n = 5. **J** Quantitative analysis of the migration rate of HUVECs. (Data are presented as the means ± SD; *p < 0.05; **p < 0.01; ***p < 0.001)

To characterize the hypo-ADSC-derived exosomal miRNAs, we analysed the expression levels of microRNAs in hypo-ADSC-Exos via high-throughput sequencing patterns with a ADSC-Exos as controls. We demonstrated that the specific miRNA signature in hypo-ADSC-Exos that was completely different from that of ADSC-Exos. The hsa-miR-1-3p was shown to be the highest level among the total miRNAs in the hypo-ADSC-Exos, followed by the hsa-miR-21-5p (Fig. 3A, B). Moreover, we found that the miR-21 was positively associated with angiopoiesis as reported previously. Then, the results were further confirmed by qRT-PCR, the expression of miR-21-5p in the hypo-ADSC-Exo group was significantly increased in HUVECs compared with the other groups (Fig. 3C). This result confirmed that an amount of mature miR-21-5p sufficient to perform biological functions was transported into the HUVECs by the ADSC-Exos. To investigate the transfer type of micro-RNA, the expression of miR-21-5p in conditioned medium (CM) was measured after treated with 10 μM GW4869 (an exosome release inhibitor) for 48 h. As shown in Fig. 3D, the levels of miR-21-5p in the CM collected from hypo-ADSCs and ADSCs treated with GW4869 were significantly decreased compared with those in the CM obtained from control hypo-ADSCs. However, there was no significant statistical difference in the ADSC group (Additional file 1: Fig. S2A). In addition, the expression of miR-21-5p in HUVECs treated with hypo-CM was also examined. The results showed that, the expression of miR-21-5p in HUVECs treated with hypo/GW4869-CM was also significantly decreased compared to that of HUVECs treated with hypo-ADSC-CM (Fig. 3E), there was also no significant statistical difference in the ADSCs group (Additional file 1: Fig. S2B), indicating that exosomes mediated the miRNA transport between the hypo-ADSCs and HUVECs.

All phenotypic experiments were conducted with four groups: the hypo-ADSC-Exo groups, hypo-ADSC-Exo+anti-miR-NC, hypo-ADSC-Exo+anti-miR-21, and a blank group (HUVECs with PBS). Previous reports demonstrated that miR-21-5p has an effect on angiogenesis and tissue regeneration. The CCK-8 and EDU assays showed that exosome stimulation resulted in a significant increase in HUVEC proliferation, and the effect was reduced by the miR-21-5p inhibitor (Fig. 3F and Additional file 1: Fig. S3). The tube formation assay was performed to obtain direct evidence of the angiogenic function of miR-21-5p on HUVECs. The results showed that there were newly growing branch points and tube lengths in the hypo-ADSC-Exo group and hypo-ADSC-Exo+anti-miR-NC group, while the tube formation in the group with inhibited miR-21-5p was weak. Thus, these results directly demonstrated the angiogenic role of hypo-ADSC-Exos in HUVECs, which could be suppressed by the miR-21-5p inhibitor (Fig. 3G, H). To determine whether exosomal miR-21-5p modulates cell migration, the wound width in the culture of each group was photographed respectively at 8 h, 16 h and 24 h. We observed that at the same time point (8 h, 16 h and 24 h), the migration rate of HUVECs in the hypo-ADSC-Exo+anti-miR-21-5p group was significantly lower than that of the other two groups (hypo-ADSC-Exo and hypo-ADSC-Exo+anti-miR-NC) and similar to that of the blank control group (Fig. 3I, J). Above all, these results demonstrated that, the hypo-ADSC-Exos promote angiogenesis in vitro via the secretion of miR-21.

### Exosomal miR-21-5p derived from hypo-ADSCs activates PI3K/AKT signalling by targeting SPRY1 in HUVECs

**Fig. 4:**
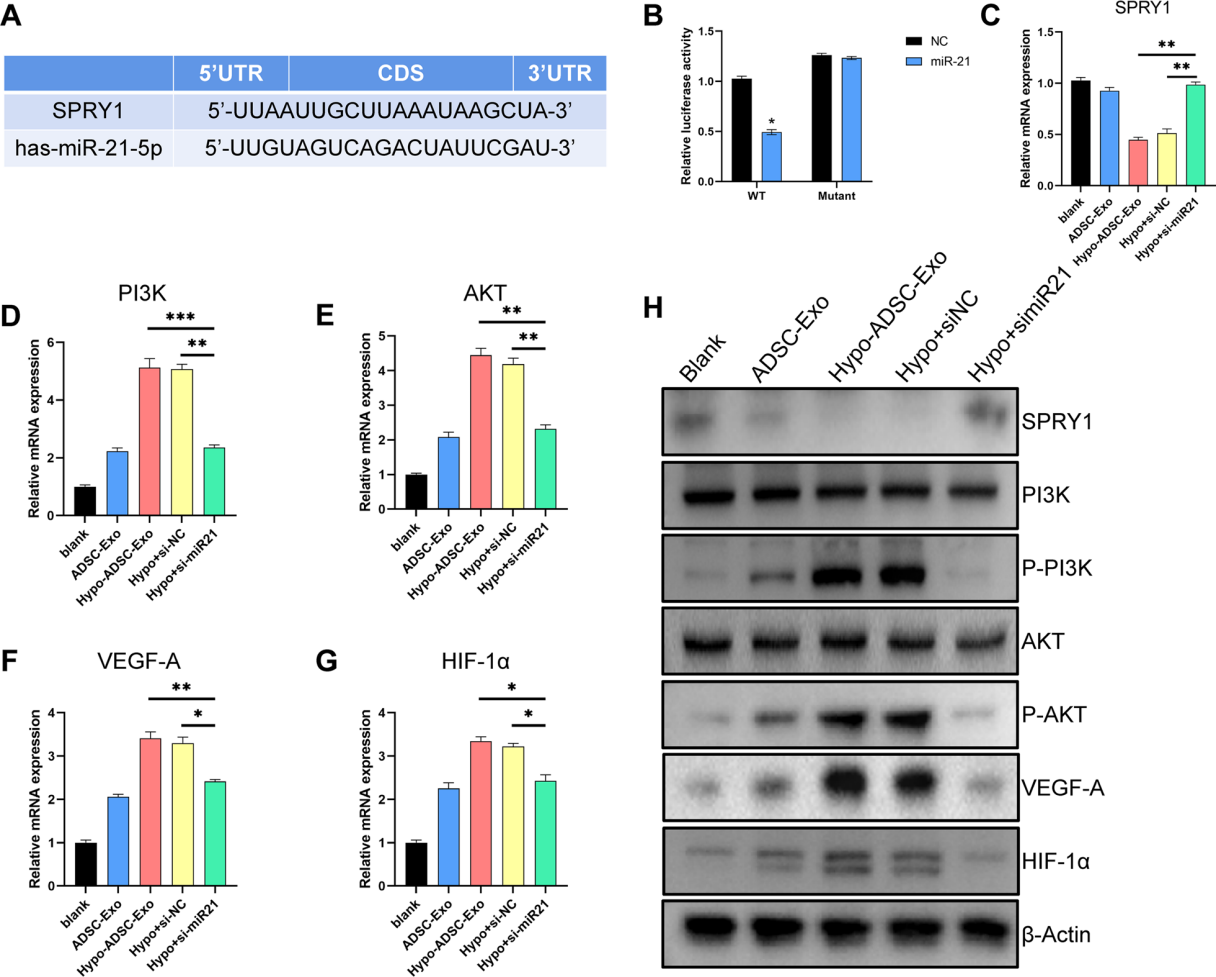
Exosomal miR-21 derived from hypoxic ADSCs activates PI3K/AKT signalling by targeting SPRY1 in the HUVECs. **A** Schematic representation of the putative binding sites for miR-21-5p in the SPRY1 3′-UTR. Predicted consequential pairings of the target regions and miR-21 (underlined) were based on data from TargetScan (www.targetscan.org). **B** Luciferase reporters containing wild-type or mutant 3′-UTR of SPRY1 genes were co-transfected with miR-21-NC or miR-21 mimics into HEK293 cells. Two days after transfection, the dual luciferase activity was measured. **C**–**G** qRT-PCR was performed to measure the mRNA level of SPRY1, PI3K, AKT VEGFA and HIF-1α in the HUVECs in different groups. **H** Western blot was performed to measure the level of SPRY1, PI3K, AKT VEGFA and HIF-1α in the HUVECs in different groups

The role of miR-21-5p in hypo-ADSC-Exos in HUVEC-mediated angiogenesis has not yet been reported, and the signalling pathways are also unknown, yet. SPRY1, which negatively regulates angiogenesis, was predicted to be a potential target gene of miR-21-5p by the microRNA (http://www.microrna.org/) and TargetScan (http://www.targetscan.org/) databases (Fig. 4A). The luciferase reporter assay results showed that miR-21-5p can bind to the 3′-UTR of SPRY1 and suppress the transcription of SPRY1 when the miR-21-5p overexpression vector was transfected into HEK293 cells (Fig. 4B).

An inhibitory effect of SPRY1 on angiogenesis, which is induced by PI3K/AKT activation in cancer cells, has been demonstrated previously [27, 28]. Thus, the miR-21-5p was knocked down to explore the downstream pathway in HUVECs. The qRT-PCR results indicated that the mRNA level of SPRY1 decreased by hypo-ADSC-Exo treatment, and minimal change was observed in the ADSC-Exo group. However, the SPRY1 increased dramatically by approximately two-fold when anti-miR-21-5p was added to interfere with HUVECs (Fig. 4C). The mRNA level of the downstream pathway P13K/AKT showed the opposite trend under the same conditions (Fig. 4D, E). Furthermore, we demonstrated that the miR-21-5p inhibitor markedly decreased the upregulated mRNA levels of HIF-1α and VEGFA (genes related to vascularization) induced by hypo-ADSC-Exos (Fig. 4F, G). These results were consistent with those of the Western blot analysis (Fig. 4H and Additional file 1: Fig. S4).

Hence, we suggest that hypo-ADSC-Exo-miR-21-5p suppresses the expression of SPRY1 and promotes the hyperactivation of AKT (p-AKT), leading to increased angiogenesis by regulating the SPRY1/PI3K/AKT signalling axis in the HUVECs. Thus, the activation of SPRY1/PI3K/AKT pathways may be the underlying mechanism by which miR-21-5p containing ADSC-Exos enhance angiogenesis.

### SPRY1 knockdown increases the HUVECs proliferation, migration and angiogenesis

**Fig. 5:**
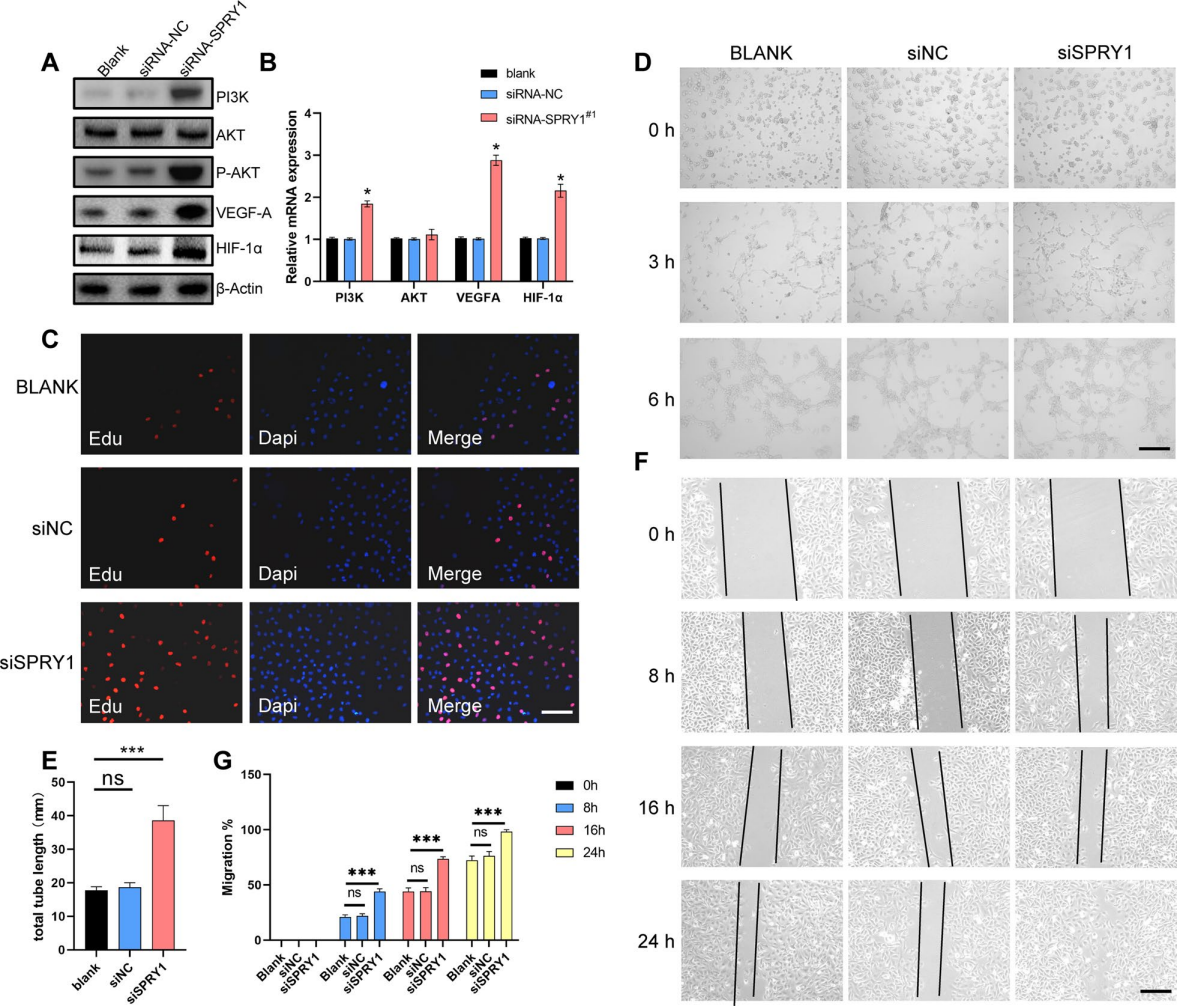
SPRY1 knockdown increases the HUVECs proliferation, migration and angiogenesis. **A**, **B** After HUVECs were transfected with SPRY1, siRNA #1 was stimulated with hypo-ADSC-Exos for 24 h and the PI3K/AKT activation levels were determined by Western blot (**A**) and qRT-PCR (**B**). **C** Cell proliferation of HUVECs measured by EDU staining. Scale bar = 200 μm. n = 5. **D** Representative images of tube formation in HUVECs treated with PBS, Hypo-Exos+si-miR-NC and Hypo-Exos, Hypo-Exos+si-miR-SPRY1. Scale bar = 200 μm. n = 5. **E** Quantitative analysis of the tube formation assay in different groups. **F** Extracted exosomes promoted cell migration as determined by a scratch test at 0 h, 8 h, 16 h and 24 h after treatment. n = 5. **G** Quantitative analysis of the migration rate of HUVECs. (Data are presented as the means ± SD; *p < 0.05; **p < 0.01; ***p < 0.001)

To confirm the key role of SPRY1 in angiogenesis and assess whether knocking down the expression of SPRY1 can achieve similar effects as ADSCs-Exos on angiogenesis. We used siRNA to inhibit the expression of SPRY1 in HUVECs. HUVECs were transfected with SPRY1 siRNAs in culture for 24 h. PI3K/AKT activation and the downstream genes (VEGFA and HIF-α) were determined by qRT-PCR and Western blot. As expected, we observed an increased expression levels of PI3K, AKT, VEGFA and HIF-1α in the ADSC-Exo+siRNA-SPRY1 group, and SPRY1 was proven to inhibit the activation of PI3K/AKT signalling pathway (Fig. 5A, B and Additional file 1: Fig. S5).

The CCK-8 and EDU assays showed that exosome stimulation resulted in a significant increase in HUVEC proliferation, and the effect was reduced by using the SPRY1 inhibitor (Additional file 1: Fig. S6 and Fig. 5C). The tube formation assay was performed and the results showed that there were newly growing branch points and tube lengths by using the SPRY1 inhibitor. These results directly demonstrated the angiogenic role of hypo-ADSC-Exos in HUVECs, which could be promoted by the SPRY1 inhibitor (Fig. 5D, E). To determine whether exosomal SPRY1 modulates cell migration, the wound width in the culture of each group was photographed respectively at 8 h, 16 h and 24 h. We observed that at the same time point (8 h, 16 h and 24 h), the migration rate of HUVECs in the siSPRY1 group was significantly higher (Fig. 5F, G). Above all, these results demonstrated that, the hypo-ADSC-Exos promote the HUVEC proliferation, migration and angiogenesis in vitro via targeting the SPRY1.

### GleMA loaded with hypo-ADSC-Exos enhanced local microvascular network formation and osteoporotic fracture healing in vivo via targeting SPRY1

**Fig. 6:**
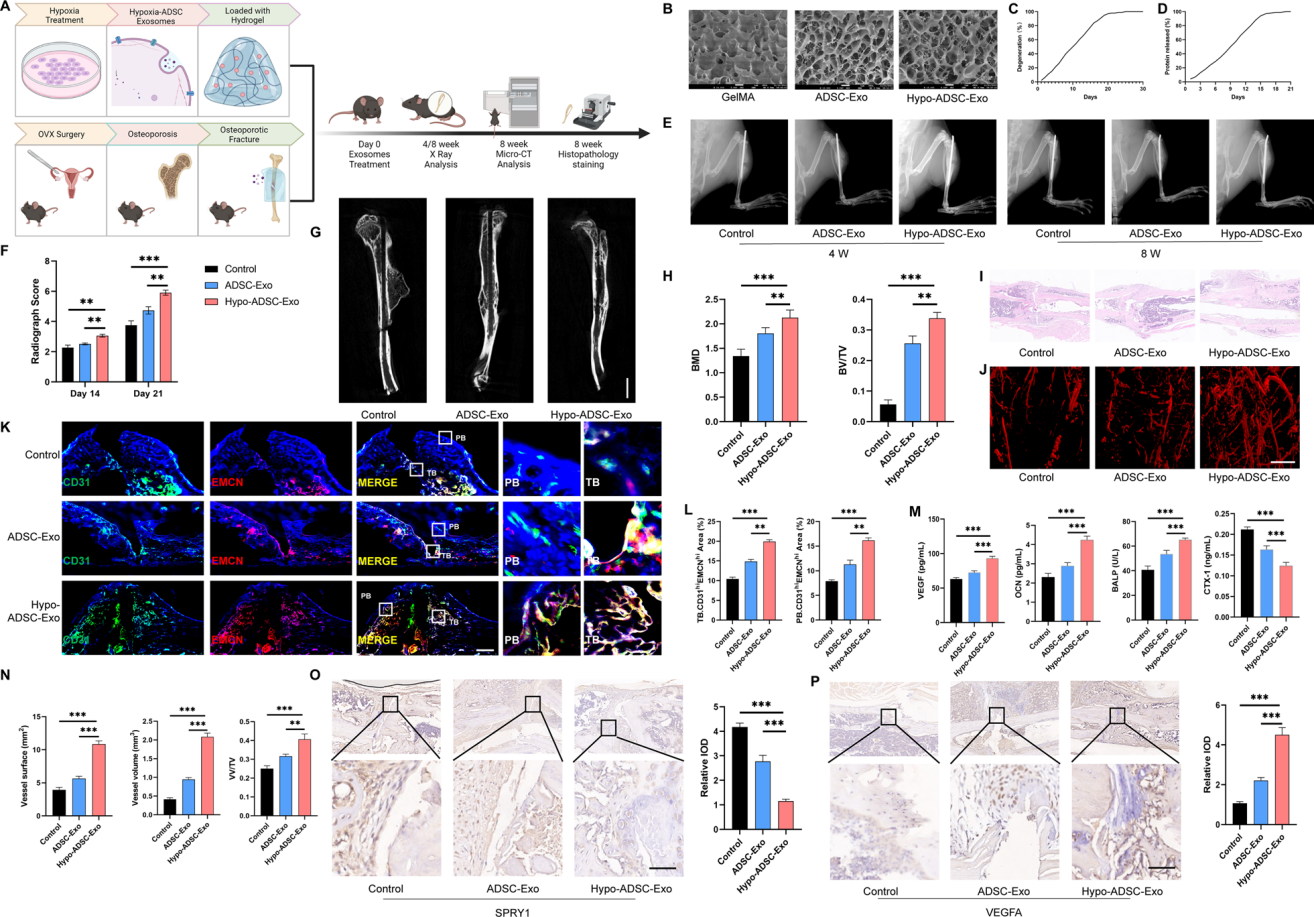
GleMA loaded with hypo-ADSC-Exos enhanced local microvascular network formation and osteoporotic fracture healing in vivo via targeting SPRY1. **A** Schematic illustration of the process of in vivo treatment using hypoxia-pretreated ADSC-derived exosomes. **B** SEM images of GelMA loaded with different exosomes. **C**, **D** Degration and protein released ratio of GelMA loaded with exosomes. **E**, **F** Representative X-Ray images and morphometric analysis of osteoporotic fractures after hypo-ADSC-Exo treatment on 4 week and 8 week. **G**, **H** Micro-CT images of osteoporotic fractures after hypo-ADSC-Exo treatment and morphometric analysis of new bone volume (BV), tissue volume (TV), and bone mineral density (BMD). n = 6. **I** Haematoxylin and eosin (H&E) staining on the bone fracture region after PBS, ADSC-Exo and hypo-ADSC-Exo treatment. n = 6. **J**, **N** Micro-CT 3D reconstruction images of angiographic images of fracture area after hypo-ADSC-Exo treatment. Scale bar, 20 μm. **K**, **L** Immunofluorescence staining and quantitative analysis results showed the CD31 and EMCN staining on the bone fracture region. Scale bar, 50 μm. n = 6. **M** The serum levels of VEGFA, OCN, BALP and CTX-1. n = 6. **O** SPRY1 immunohistochemistry staining images and quantitative analysis on the bone fracture region after PBS, ADSC-Exo and hypo-ADSC-Exo treatment. Scale bar, 50 μm. n = 6. **P** VEGFA immunohistochemistry staining images and quantitative analysis on the bone fracture region after PBS, ADSC-Exo and hypo-ADSC-Exo treatment. Scale bar, 50 μm. n = 6. (Data are presented as the means ± SD; *p < 0.05; **p < 0.01; ***p < 0.001)

To investigate the influence of hypo-ADSC-Exos on the repair of osteoporotic fracture in vivo, a mice osteoporotic fracture model was established for a duration of 3 months (Fig. 6A and Additional file 1: Fig. S7A). GelMA hydrogels are extensively used in tissue engineering due to their composition, resembling natural extracellular matrices with cell adhesion sites and sequence responsive to matrix metalloproteinases [25]. Initially, GelMA was synthesized via the transesterification process involving gelatin and methacrylate, and the structure of methacrylic acid was determined by ^1^HNMR and FT-IR (Additional file 1: Fig S7B, C). Subsequently, upon exposure to 405 nm blue light for 1 min, the pre-hydrogel solution underwent solidification, demonstrating the photocuring performance and the malleability of the hydrogel characteristics (Additional file 1: Fig. S7D). To create GelMA-Exo hydrogels, exosomes were introduced into the GelMA hydrogel pre-solution and exposed to 405 nm blue light. The resulting hydrogels, incorporating exosomes were examined using SEM, degradation and protein released analyses (Fig. 6B–D). It’s evident that the hydrogels maintained a porous and loose structure post the incorporation of exosomes, the pore walls contained numerous nano-sized exosome particles.

Then, X-ray was used to evaluate the extent of osteoporotic bone fracture repair in three groups. The administration of hypo-ADSC‐Exos loaded in GelMA led to an obvious increase in the amount of new bone formation compared with that in the control and ADSC-Exo groups (Fig. 6E, F). Moreover, the micro-CT showed that, the hypo-ADSC-Exo treatment led to a significant increase in the bone volume/tissue volume BV/TV and bone mineral density (BMD) (Fig. 6G, H). H&E staining was used to observe the microscopic and newly formed bone. The hypo-ADSC-Exos treatment group showed better new bone formation around the osteoporotic fracture area (Fig. 6I). Vascular growth within the bone fracture region was evaluated by imaging of contrast‐perfused, decalcified specimens. The vessel volume was remarkably increased in the hypo-ADSC-Exo treatment group (Fig. 6J, G). CD31^hi^EMCN^hi^ vessels were proven to be coupled with angiogenesis and osteogenesis during osteoporotic fracture healing [7]. Therefore, we performed CD31 and EMCN immunofluorescence staining to explore the formation of CD31^hi^EMCN^hi^ vessels on both trabecular bone (TB) and periosteal bone (PB). The results showed that, the formation of CD31^hi^EMCN^hi^ vessels was more abundant in the hypo-ADSC-Exo group, than in the control and ADSC-Exo groups in both TB and PB (Fig. 6K, L). We further evaluated the condition of angiogenesis and osteogenesis after hypo-ADSC-Exos treatment by detecting the serum levels of VEGFA, OCN, BALP and CTX-1. The serum VEGFA level was up-regulated after hypo-ADSC-Exos treatment compared with that in control and ADSC-Exos group, which represented a stronger angiogenic effect in the hypo-ADSC-Exos group. The serum-increased levels of OCN and BALP proved that hypo-ADSC-Exos enhanced the bone regeneration and the serum-decreased levels of CTX-1 proved the hypo-ADSC-Exos inhibited bone resorption (Fig. 6M).

In order to explore the mechanisms of the hypo-ADSC-Exos on bone regeneration, the expression of SPRY1 and VEGFA were analyzed by immumohistochemical staining. The relative integrated optical density (IOD) value of the SPRY1 around the osteoporotic fracture area was significantly decreased after hypo-ADSC-Exo treatment (Fig. 6O). In addition, the expression of VEGFA was increased, which was consistent with our in vitro results (Fig. 6P). In conclusion, these results were consistent with the results from our cell experiments, implying that exosomal miR-21-5p delivery inhibited the expression of SPRY1 and increased the CD31^hi^EMCN^hi^ vessel formation in the TB and PB regions, which enhanced bone formation in the osteoporotic fracture space.

## Discussion

Previous study confirmed that exosomes derived from ADSCs are beneficial to wound and fracture healing in both a cell culture model and a well-established clinically relevant model [23, 29]. Interestingly, we found that hypo-ADSC-Exos played a more important angiogenic role in the process of osteoporotic fracture healing, but not only in the proliferation of osteoblasts described previously [30]. In the field of osteoporotic fracture healing, one of the most critical factors for union is sufficient blood supply at the fracture site [7, 31]. Angiogenesis is observed before osteogenesis and continues throughout the entire healing process [29]. Thus, promoting angiogenesis immediately after trauma is urgent and even critical. Therefore, in this paper, we provide the demonstration of the mechanism underlying the ability of GelMA-hypo-ADSC-Exos to promote angiogenesis.

As intercellular communication is required for various physiological and pathological processes, exosomes can be readily isolated from stem cells from various origins, and these exosomes carry biologically active molecules (protein, microRNA, and DNA) that can be transferred to target cells to exert therapeutic effects [32, 33]. Current research has demonstrated that the hypoxic pre-treatment could promote the tissue healing [34–36]. Herein, we analysed the global expression of miRNAs in hypo-ADSC-Exos via high-throughput sequencing and miR-21-5p was found in greater abundance in the hypo-ADSC-Exo than it was in the control group. Moreover, the expression of miR-21-5p in the HUVECs was significantly increased following treatment with hypo-ADSC-Exos. The immunofluorescence results indicated that exosomal miR-21-5p was indeed transferred to the HUVECs. As reported in the literature, miR-21-5p is frequently overexpressed in human cancers and acts as an oncogene, and it also plays a regulatory role in endothelial cell proliferation and migration and influences angiogenesis by interacting with hypoxia inducible factor 1 alpha (HIF-1α) or other factors [37–39]. Thus, in this study, we hypothesize that exosomal miR-21-5p, the most abundant miRNA in the hypo-ADSC-Exos, was the main transmitter of cellular information.

To confirm the role of exosomal miR-21-5p in this process, we transfected an inhibitor of miR-21-5p into hypo-ADSCs, extracted the exosomes, and determined the expression level of miR-21-5p in these exosomes. The expression decreased correspondingly, and subsequent tests showed a trend of proliferation and migration suppression when the HUVECs were co-cultivated with hypo-ADSC-Exos with a miR-21-5p inhibitor. Finally, the pro-angiogenesis function of HUVECs was also confirmed, and the results showed that exosomal miR-21-5p from ADSCs played a crucial angiogenic role in the HUVECs. However, details on the downstream pathway remain to be provided.

Bioinformatics analyses and luciferase assays were used to confirm that miR-21-5p binds directly to the 3′-UTR of the SPRY1 mRNA and inhibits the transcription of SPRY1. SPRY1 was previously described as a potent angiogenesis inhibitor in endothelial cells and was found to inhibit the ERK/MAPK signaling induced by bFGF and VEGF [40–42]. Recent studies have shown that miR-21-5p contributes to the proliferation, migration, and apoptosis of cancer cells with the concomitant degradation of SPRY1 [41, 43]. However, few studies have focused focused on the mechanism by which miR-21-5p targets of SPRY1 during angiogenesis or fracture healing. Our qRT-PCR and Western blot data showed that the mRNA level of SPRY1 increased dramatically when hypo-ADSC-Exos with miR-21-5p inhibitor were transfected into HUVECs. The mRNA expression of VEGFA and HIF-1α and the phosphorylation of AKT in the HUVECs were significantly decreased. It is reported that VEGFA plays an important role in the endothelial cell mitosis and blood vessels fusion, thereby promoting neovascularization. And HIF-1α exhibits enhanced stability under hypoxic conditions and regulates VEGFA expression through transcriptional activation [37, 44, 45]. AKT is a well-characterized target of PI3K, and AKT phosphorylation is essential for the activation of the PI3K/AKT pathway. In the context of angiogenesis, accumulating evidence from a number of studies has shown that the PI3K/AKT signalling pathway plays a key role in ischaemic injury and the expression of multiple angiogenic molecules, such as VEGF and SDF-1, may be upregulated in this pathway [46]. To confirm that angiogenesis depends on SPRY1, we also used siRNA to inhibit the expression of SPRY1 in HUVECs. The qRT-PCR and Western blot analysis results showed that SPRY1 siRNA significantly inhibited the expression of HIF-1α and VEGF as well as PI3K/AKT pathway components. In addition, a series of results on cell function suggested that the angiogenic ability of HUVECs had been restricted. In accordance with these findings, the miR-21-5p-dependent regulation of endothelial cell activity and angiogenesis were regulated by the activation of PI3K/AKT signalling upon downregulation of SPRY1.

The osteoporotic fracture model is commonly used for studying bone formation in aging. Controlled released of exosomes from GelMA hydrogels provide a potential approach on bone repair. To make our experimental results more convincing, we studied micro-vessels and bone formation in vivo. The role of hypo-ADSC-Exo loaded in GelMA was evaluated by testing its ability in osteoporotic fracture repair in a mouse model. The restoration of blood flow is critical in bone formation not only for the transport of oxygen and nutrients but also for the initiation of ossification and recruitment of MSCs. Therefore, it was not surprising that the hypo-ADSC-Exo loaded in GelMA induced formation of micro-vessel networks promoted osteoporotic bone healing. A specific vascular subtype in bone termed type H vessels, characterized by strong positivity for both CD31 and Endomucin (Emcn) in the endothelium, has recently been shown to couple angiogenesis with osteogenesis [7]. In the current model, newly formed vessels in the fracture area were observed more clearly through three‐dimensional images of the radiopaque contrast‐filled vascular network. Moreover, the results from the in vivo study not only verified our hypothesis that an SPRY1-dependant mechanism induces angiogenesis but also indicated that the trends of significant in vivo microvascular remodelling and osseous regeneration lend substantial credibility to the utilization of hypo-ADSC-Exos and exosomal miR-21-5p in the development of future therapeutic tissue remodelling strategies.

## Conclusions

**Fig. 7:**
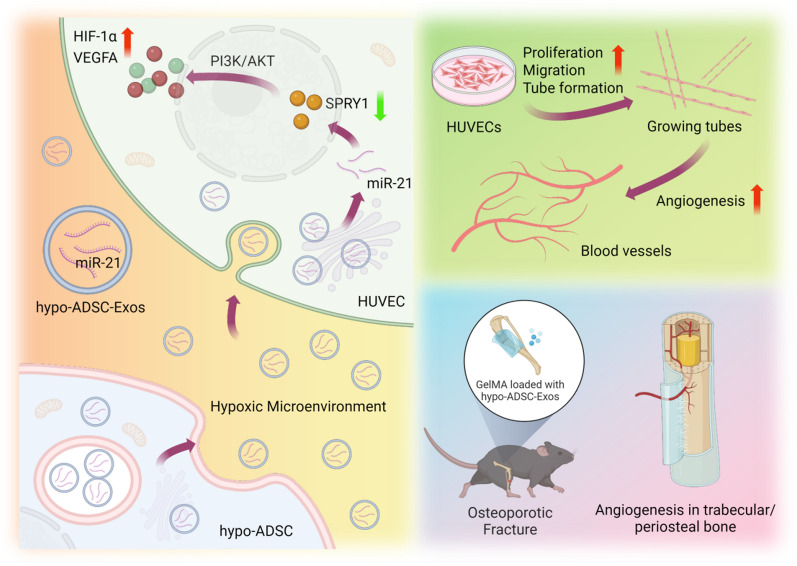
Schematic representation of proposed hypo-ADSC-Exo mechanism of action in osteoporotic bone repair regeneration

Taken together, the findings from our current study indicate that the specific miR-21-5p, which is shuttled directly from hypo-ADSCs to HUVECs via exosomes, regulates HUVEC proliferation, migration and tube formation through the activation of the PI3K/AKT pathway and induction of HIF-1α and VEGFA expression by suppressing the target gene SPRY1. Furthermore, an osteoporotic fracture model was utilized to verify this pro-angiogenic phenomenon and its impact on bone formation (Fig. 7). Overall, the data presented herein provide the first evidence of a novel mechanism of hypo-ADSC-Exos in inducing angiogenesis, which may provide a basis for prospective therapeutic approaches in the treatment of atrophic delayed or non-union osteoporotic fracture in the future.

## Materials and methods

### Hypo-ADSCs cultures

ADSCs were cultured in α-MEM supplemented with 10% FBS and 1% penicillin as well as 1% streptomycin (Gibco BRL, Grand Island, USA). The cell culture protocols were authorized by the Ethical Committee of the Navy Medical University. For hypoxic treatment of ADSCs, the cells were incubated in a hypoxic environment (1% O_2_) for 24 h. In total, samples from three different healthy donors were collected successively to obtain exosomes.

### Quantitative real-time PCR

Total RNA from HUVECs transfected with ADSC-Exo-anti-miR-NC, ADSC-Exo-anti-miR-21-5p and ADSC-Exo was isolated by TRIzol reagent (Thermo Fisher Scientific Inc., Waltham, MA, USA) according to the manufacturer’s instructions. The concentration and purity of the total RNA were measured by a Thermo Scientific™ NanoDrop™ spectrophotometer (Thermo Fisher Scientific). Then, first-strand cDNA was reverse-synthesized from 2 μg of total RNA with a cDNA synthesis kit (Thermo Fisher Scientific Inc.). Quantitative real-time PCR (qRT-PCR) was performed in a 25-μL reaction volume. The primers were shown in Table 1.

**Table 1:** The qPCR primers used in the research

Gene	Sequence	
qPCR primers		
hsa-miR-1-3p	F	TAAAGTGGGGACAGCAAAATGC
hsa-miR-1-3p	R	AGCACAAGGTAGAGAAGGTAGAG
hsa-miR-21-5p	F	ACACTCCAGCTGGGTAGCTTATCAGACTGA
hsa-miR-21-5p	R	CTCAACTGGTGTCGTGGAGTCGGCAATTCAGTTGAGTCAACATC
hsa-miR-143-3p	F	GCCAGAGCTGGAGAGGTGGAG
hsa-miR-143-3p	R	AACTGACCAGAGATGCAGCACTG
hsa-let-7f-5p	F	CGCGCGTGAGGTAGTAGATTGT
hsa-let-7f-5p	R	AGTGCAGGGTCCGAGGTATT
hsa-miR-199a-3p	F	AGCTTCTGGAGATCCTGCTCC
hsa-miR-199a-3p	R	TCCCTTGCCCAGTCTAACCAA
hsa-miR-26a-5p	F	UCCAUAAAGUAGGAAACACUACA
hsa-miR-26a-5p	R	CAGUACUUUUGUGUAGUACAA
hsa-miR-451a	F	ACACTCCAGCTGGGAAACCGTTACCATTACT
hsa-miR-451a	R	CTGGTGTCGTGGAGTCGGCAA
hsa-let-7i-5p	F	UGAGGUAGUAGUUUGUGCUGUU
hsa-let-7i-5p	R	CAGCACAAACUACUACCUCAUU
hsa-miR-148a-3p	F	GGTCAGTGCACTACAGAACTTTG
hsa-miR-148a-3p	R	GATGATGATAAGCAAATGCTGACTGAAC
hsa-miR-100-5p	F	GGAACCCGTAGATCCGAACTTGTG
hsa-miR-100-5p	R	AACGCTTCACGAATTTGCGT
hsa-miR-146a-5p	F	GGCGGTGAGAACTGAATTCC
hsa-miR-146a-5p	R	CGCAGTAGCACCATTTGAAAT
hsa-miR-199b-3p	F	CCAGAGGACACCTCCACTCC
hsa-miR-199b-3p	R	GGGCTGGGTTAGACCCTCGG
hsa-miR-99a-5p	F	AACCCGTAGATCCGATCTTGTG
hsa-miR-99a-5p	R	CACAAGATCGGATCTACGGGTT
hsa-let-7g-5p	F	GGGTGAGGTAGTAGTTTGT
hsa-let-7g-5p	R	CAGTGCGTGTCGTGGAGT
hsa-miR-30a-5p	F	AACGAGACGACGACAGAC
hsa-miR-30a-5p	R	TGTAAACATCCTCGACTGGAAG
β-Actin	F	AGAGCTACGAGCTGCCTGAC
β-Actin	R	AGCACTGTGTTGGCGTACAG

### Purification and identification of exosomes

First, the ADSCs/hypo-ADSCs were seeded at 1.5 × 10^5^ cells in a T25 polystyrene culture flasks (Corning) with 5 mL of α-MEM culture medium for 24 h adherence. After that, the flasks were washed for 3 times with PBS and the culture medium with exo-free-FBS [47] was added in them. After 48 h, the freshly collected supernatant was centrifuged at 1500×*g* for 5 min at room temperature and then 10,000×*g* for half an hour at 4 °C to remove the impurities. Exosomes were precipitated by an ExoQuick-TC kit (ExoQuick-TC) following by the manufacturer’s instructions. Then, we added PBS to re-suspend the exosomes, which were stored in a freezer at − 80 °C for use in subsequent bioassays. A BCA kit (Thermo Fisher Scientific Inc.) was used to detect the concentration levels of exosomes. The isolated exosomes were fixed with 4% PFA, deposited onto electron microscopy grids according to well-established consecutive procedures, and directly photographed with a transmission electron microscope (Tokyo, Japan) at 80 kV. Then, the identity of the exosomes was determined or confirmed by their binding affinity for CD63, CD81, and TSG101 antibodies (Abcam, USA), which were reported to be specific exosome biomarkers [48].

### Characterization of GelMA-Exo hydrogels

The surface characteristics of GelMA and GelMA-Exo hydrogels were examined using a scanning electron microscope (SEM). For the analysis of biodegradability ratio, the hydrogels’ initial mass was determined. Subsequently, the hydrogels were placed in a 2 U/mL collagenase PBS for incubation at 37 °C. The ongoing mass were monitored over time.

### RNA interference

The miR-21-5p inhibitor sequence was (5′-UCAACAUCAGUCUGAUAAGCUA-3′). The siRNA-SPRY11 SS: GACACUUGUGCAUAAGUUAUC, AS: UAACUUAUGCACAAGUGUCAG, siRNA-SPRY12 SS: GGUCUGUCGUGCUAAUAAAUG, AS: UUUAUUAGCACGACAGACCUU.

The sequence of the negative control oligonucleotide (5′-CAGUACUUUUGUGUAGUACAA-3′) was provided by RiboBio (Shanghai, China). Then cell transfection process was performed following the manufacturer’s instructions.

### Luciferase reporter assay

We predicted the potential binding sites of miR-21-5p to obtain fragments sequences on the biological prediction website (http://www.microrna.org), and the SPRY1 was proven to be as a target gene to miR-21-5p. Chemical synthesis was performed, after that, luciferase reporter plasmid was cotransfected into HEK293 cells. Cells were incubated for 36 h in a 12-well plate. After transfection, the cells were examined by a commercial dual-luciferase reporter assay kit (Nanjing, Jiangsu, China), and the luciferase activity was detected.

### Western blotting

HUVECs in different groups were washed with ice-cold PBS three times after the culture medium was removed. The total protein content was extracted from the HUVECs by highly concentrated RIPA lysis buffer and detected by the BCA protein assay kit. 10 μg of total protein was added to SDS-PAGE and transferred into a PVDF membrane. A 5% non-fat milk solution prepared with 1×TBST was utilized to block the PVDF membrane for one hour at 4 °C. Then, the PVDF membrane was incubated with the following specific primary antibodies for 1 h at room temperature: anti-PI3K (1:1000, CST, USA), anti-SPRY1 (1:1000, CST, USA), anti-VEGFA (1:1000, CST, USA), anti-HIF-1α (1:1000, CST, USA), anti-p-AKT (1:1000, CST, USA), anti-AKT (1:1000, CST, USA), anti-β-actin (1:5000, CST, USA), anti-CD9 (1:1000, CST, USA), anti-CD81 (1:1000, CST, USA) and anti-CD63 (1:1000, CST, USA). The reaction was captured on BioMax film (Kodak, Rochester, NY, USA). β-Actin or CD9 was used as internal control.

### Cell proliferation assay

To put it simply, 1 × 10^5^ HUVEC cells/well were incubated in a 24-well plates and treated with ADSC-Exos (10 μg/well), ADSC-Exo-anti-miR-NC and ADSC-Exo-anti-miR-21-5p. A group of HUVECs with the same volume of PBS served as the control group. At different points in time, 15 μL of CCK-8 solution (Kyushu Island, Japan) was added to each well. Cells were incubated at room temperature for 2 h. The absorbance of cells was detected at 450 nm by a microplate reader.

### Tube formation assay (in vitro angiogenesis)

Cryopreserved HUVECs were seeded in a tissue culture flask. A commercial tube formation assay kit (Cell Biolabs, USA) was used to assess in vitro angiogenesis within 18 h of cell seeding. Thawed ECM gel was added to a plate and cultured for 1 h. After that, HUVECs in suspension were added to the solidified ECM gel and cultured for 12 h at room temperature. We examined and imaged the tube formation at 12 h and 24 h using a light microscope in a high magnification field.

### Scratch test

A total of 2 × 10^5^ HUVECs were placed into a 12-well plate and incubated until confluence. Using pipette tip, we made a straight scratch in the monolayer to simulate a wound. After 0 h, 6 h, 12 h and 24 h of incubation, the width of the wound was photographed and cell migration was evaluated using an optical microscope (Leica, Germany). The width of the wound at 0 h was set as a negative control. Moreover, the wound width at each time point was normalized to that at 0 h and compared to the width measured at other time points. The assay was repeated at least three times.

### Osteoporotic fracture model

12-week-old female mice were carried out for ovariectomy surgery and randomly assigned to 3 groups. All surgical procedures were performed under general anaesthesia using intraperitoneal injection of pentobarbital sodium (4%, 9 mL/kg body weight), and post-operative analgesic care was ensured with the administration of tramadol. Then, the osteoporotic mice were used for a osteoporotic tibia fractures model. A fracture model was created in the mid-tibia using a rongeur. All operations were performed under sterile conditions. All surgeries were performed according to a protocol approved by the Institutional Animal Care Committee at the Navy Medical University.

### Micro-CT analysis

The mice were sacrificed successively at predetermined time points over 8 weeks. The bone of each mice was obtained and fixed in a 4% paraformaldehyde solution before further analysis. The vascularity was detected using a micro‐CT‐based method as described previously. The fracture area was analysed to calculate the percentage of new bone volume (BV), tissue volume (TV), BV/TV, vessel volume (VV), and bone mineral density (BMD) (n = 6).

### Histological analysis

Specimens and intact surrounding tissues were further processed to obtain 6 μm paraffin‐embedded sections. Haematoxylin & eosin (HE) staining was conducted for histological assessments. SPRY1, VEGFA, CD31 and EMCN expression was measured by immunohistochemistry with the appropriate antibodies (Abcam, USA). Ultimately, all the sections were observed with a light microscope (Leica, Germany), and then the levels of VEGFA, CD31, EMCN and SPRY1 expression were analyzed in each sample via Image J software.

### High-throughput sequencing

Briefly, RNA was isolated from exosomes released by ADSCs and hypo-ADSCs using Trizol (Life Technologies, UK). Transcriptome sequencing data (mRNA) were generated through the HiSeq 2500 platform (Illumina, USA). Differentially expressed genes (DEGs) were determined based on an absolute log2 fold change ≥ 0.5 and p < 0.05. The p-value threshold used in the investigation of DEG biological functions indicates the correlation between the screened signal pathway and the target gene. TargetScan was utilized for identifying potential targets of miR-21, and the outcomes were subsequently validated through PCR.

### Angiographic images

The method for detecting blood vessels in the femur bone using micro-CT was performed following a previously established protocol1. Mice were subjected to anesthesia using 4% chloral hydrate, and the vasculature was thoroughly rinsed with saline solution followed by two washes with 4% paraformaldehyde solution. Subsequently, another saline solution rinse was performed, and a dye (MICROFIL, MV-120, Blue, Flow Tech) was injected into the vasculature. The mice were then stored at 4 °C for 12 h, after which the tibia were extracted. The tibia underwent fixation for 3 days and decalcification for 20 days before micro-CT analysis.

### Serum assays

The serum levels of CTX-1, BALP, OCN and VEGF were examined by ELISA using a CTX-1 ELISA kit (NBP2-69074, Novus), OCN ELISA kit (NBP2-68151, Novus), BALP ELISA kit (CSB-E09033h), and VEGF ELISA kit (ab100751, Abcam). All ELISAs were performed in accordance with the manufacturer’s instructions.

### Statistical analysis

Each assay in this study was performed in triplicate. All valuable data were statistically analysed by SPSS 22.0 and presented as the mean ± SD. A two-sided Student’s *t*-test was performed to compare the differences between two groups. The difference was considered as significant when the *p* value was less than 0.05 or 0.01.

### Supplementary Information

**Additional file 1: Figure S1.** Cell proliferation of HUVECs after PBS, ADSC-Exo and hypo-ADSC-Exo administration as measured by CCK8 assay (n = 6). **Figure S2.** (A) The expression of miR-21 in the CM of ADSCs treated with or without GW4869. (B) Expression of miR-21 in the HUVECs treated with or without GW4869-CM. **Figure S3.** Cell proliferation of HUVECs after PBS, hypo-ADSC-Exo, hypo-ADSC-Exo+siNC and hypo-ADSC-Exo+si--miR-21 administration as measured by CCK8 assay (n = 6). **Figure S4.** Quantitative results in the Western blot data (n = 3). **Figure S5.** Quantitative results in the Western blot data (n = 3). **Figure S6.** Cell proliferation of HUVECs after PBS, siNC and siSPRY1 administration as measured by CCK8 assay (n = 6). **Figure S7.** The characteristics of GelMA. A. The GelMA placed on the fracture site. B. The structure of methacrylic acid was determined by ^1^HNMR. C. The structure of methacrylic acid was determined by FT-IR. D. The photocuring performance and the malleability of the hydrogel characteristics.

## Data Availability

Data and material will be deposited and publicly available.

## Abbreviations

ADSCs: Adipose derived stem cells
hypo-ADSC-Exos: Hypoxia preconditioning ADSC exosomes
GelMA: Gelatin methacryloyl
HUVECs: Human umbilical vein endothelial cells
SPRY1: Sprouly 1
HIF: Hypoxia inducible factor
VEGF-A: Vascular growth factor-A
HE: Haematoxylin & eosin
TB: Trabecular bone
PB: Periosteal bone
