# Supplementary material for: Hypoxia preconditioning of adipose stem cell-derived exosomes loaded in gelatin methacryloyl (GelMA) promote type H angiogenesis and osteoporotic fracture repair
Source: J Nanobiotechnology. 2024 Mar 15;22:112. doi: 10.1186/s12951-024-02342-6 (PMC10943905; doi:10.1186/s12951-024-02342-6)
Supplement: Supplementary file 1 — Additional file 1: Figure S1. Cell proliferation of HUVECs after PBS, ADSC-Exo and hypo-ADSC-Exo administration as measured by CCK8 assay (n = 6). Figure S2. (A) The expression of miR-21 in the CM of ADSCs treated with or without GW4869. (B) Expression of miR-21 in the HUVECs treated with or without GW4869-CM. Figure S3. Cell proliferation of HUVECs after PBS, hypo-ADSC-Exo, hypo-ADSC-Exo+siNC and hypo-ADSC-Exo+si--miR-21 administration as measured by CCK8 assay (n = 6). Figure S4. Quantitative results in the Western blot data (n = 3). Figure S5. Quantitative results in the Western blot data (n = 3). Figure S6. Cell proliferation of HUVECs after PBS, siNC and siSPRY1 administration as measured by CCK8 assay (n = 6). Figure S7. The characteristics of GelMA. A. The GelMA placed on the fracture site. B. The structure of methacrylic acid was determined by 1HNMR. C. The structure of methacrylic acid was determined by FT-IR. D. The photocuring performance and the malleability of the hydrogel characteristics. [file 12951_2024_2342_MOESM1_ESM.docx]

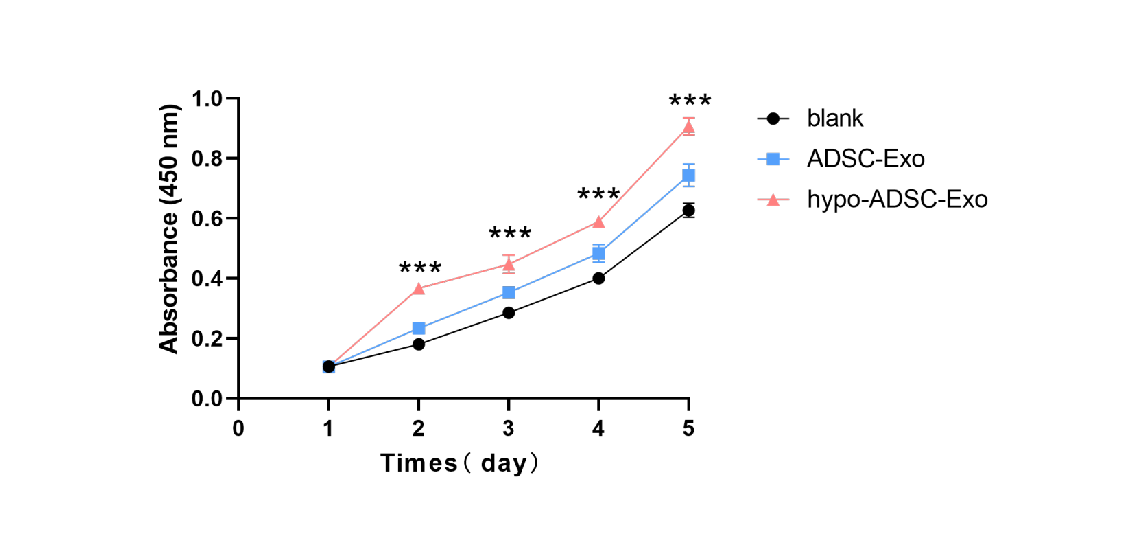


Figure S1. Cell proliferation of HUVECs after PBS, ADSC-Exo and hypo-ADSC-Exo administration as measured by CCK8 assay (n = 6).


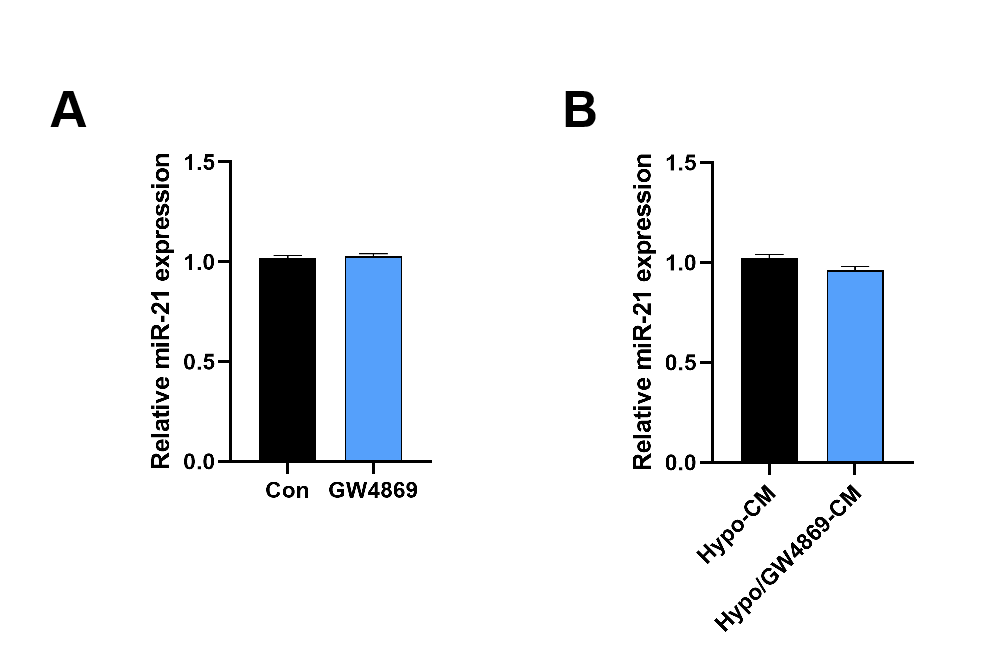


Figure S2. (A) The expression of miR-21 in the CM of ADSCs treated with or without GW4869. (B) Expression of miR-21 in the HUVECs treated with or without GW4869-CM.


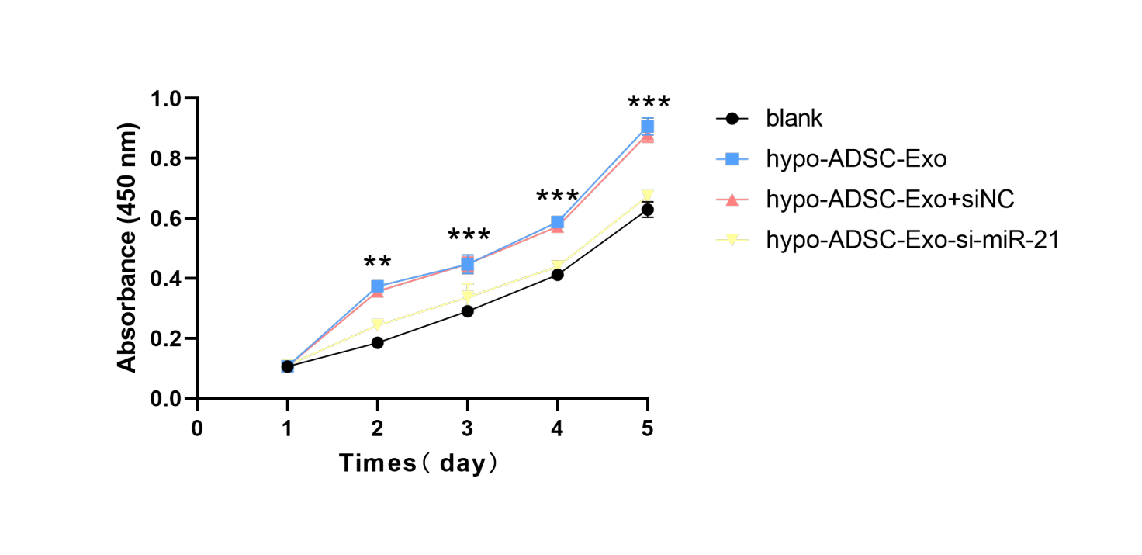


Figure S3. Cell proliferation of HUVECs after PBS, hypo-ADSC-Exo, hypo-ADSC-Exo+siNC and hypo-ADSC-Exo+si--miR-21 administration as measured by CCK8 assay (n = 6).


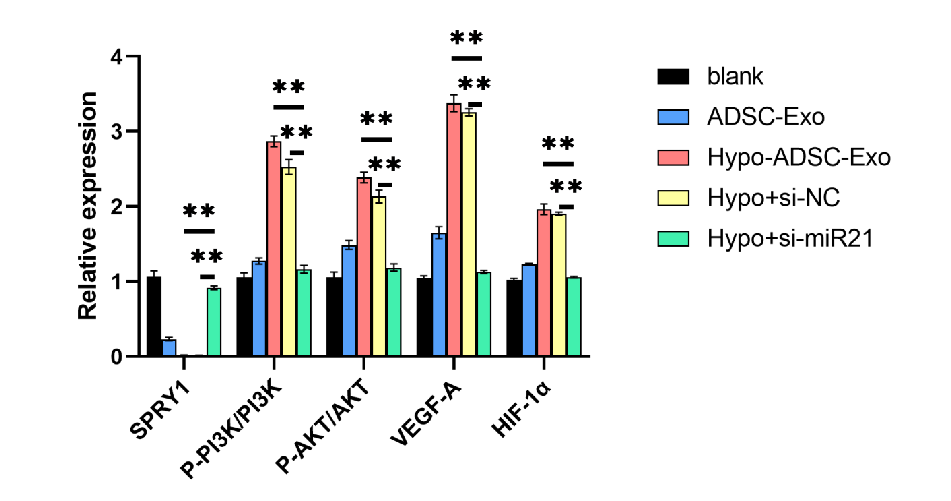


Figure S4. Quantitative results in the Western blot data (n=3).


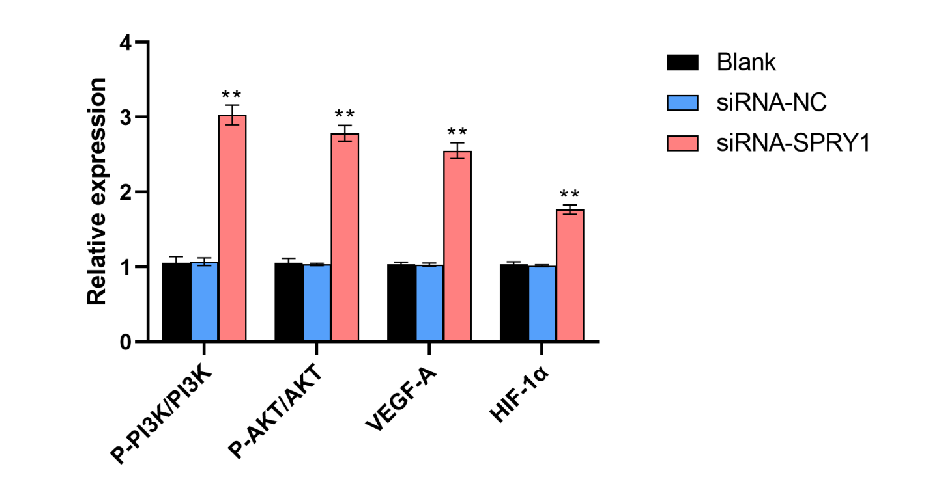


Figure S5. Quantitative results in the Western blot data (n=3).


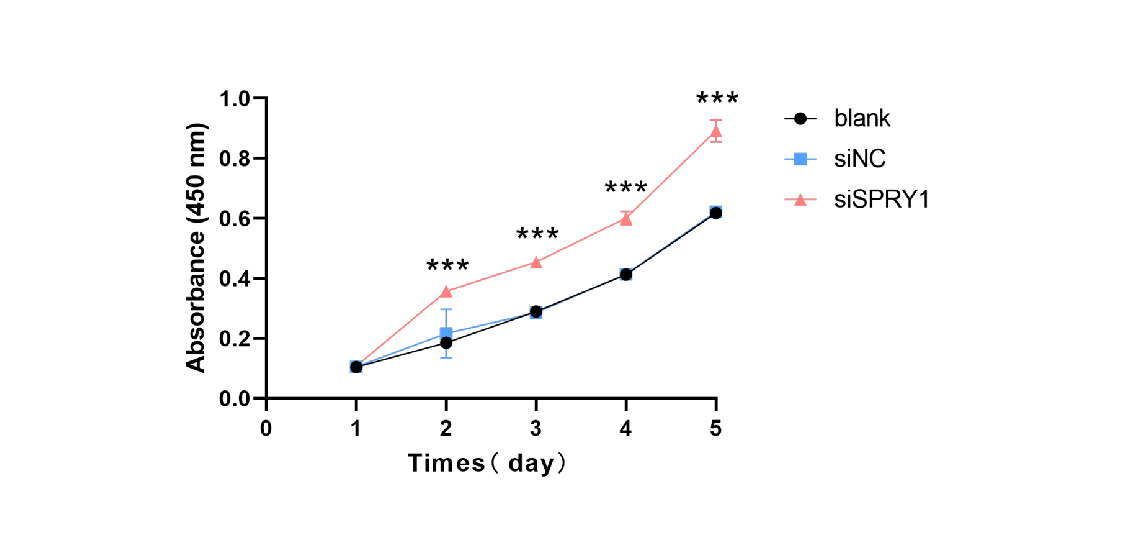


Figure S6. Cell proliferation of HUVECs after PBS, siNC and siSPRY1 administration as measured by CCK8 assay (n = 6).


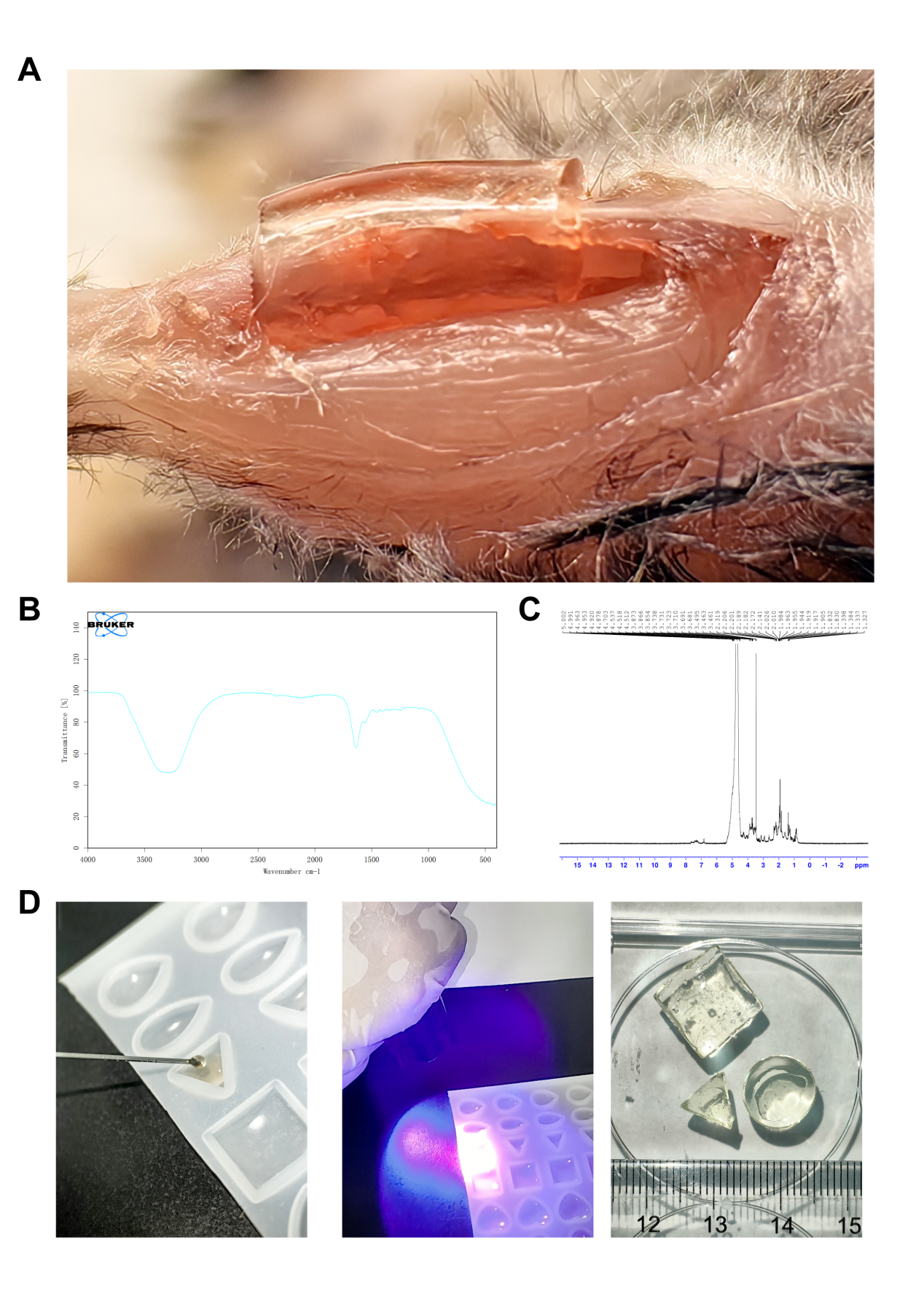


Figure S7. The characteristics of GelMA. A. The GelMA placed on the fracture site. B. The structure of methacrylic acid was determined by ^1^HNMR. C. The structure of methacrylic acid was determined by FT-IR. D. The photocuring performance and the malleability of the hydrogel characteristics.
